# In situ monitoring magnetism and resistance of nanophase platinum upon electrochemical oxidation

**DOI:** 10.3762/bjnano.4.46

**Published:** 2013-06-24

**Authors:** Eva-Maria Steyskal, Stefan Topolovec, Stephan Landgraf, Heinz Krenn, Roland Würschum

**Affiliations:** 1 Institute of Materials Physics, Graz University of Technology, Petersgasse 16, 8010 Graz, Austria; 2 Institute of Physical and Theoretical Chemistry, Graz University of Technology, Stremayrgasse 9, 8010 Graz, Austria; 3 Institute of Physics, University of Graz, Universitätsplatz 5, 8010 Graz, Austria

**Keywords:** electrical resistance, electrochemistry, magnetism, porous nanocrystalline Pt, tunable properties

## Abstract

Controlled tuning of material properties by external stimuli represents one of the major topics of current research in the field of functional materials. Electrochemically induced property tuning has recently emerged as a promising pathway in this direction making use of nanophase materials with a high fraction of electrode-electrolyte interfaces. The present letter reports on electrochemical property tuning of porous nanocrystalline Pt. Deeper insight into the underlying processes could be gained by means of a direct comparison of the charge-induced response of two different properties, namely electrical resistance and magnetic moment. For this purpose, four-point resistance measurements and SQUID magnetometry were performed under identical in situ electrochemical control focussing on the regime of electrooxidation. Fully reversible variations of the electrical resistance and the magnetic moment of 6% and 1% were observed upon the formation or dissolution of a subatomic chemisorbed oxygen surface layer, respectively. The increase of the resistance, which is directly correlated to the amount of deposited oxygen, is considered to be primarily caused by charge-carrier scattering processes at the metal–electrolyte interfaces. In comparison, the decrease of the magnetic moment upon positive charging appears to be governed by the electric field at the nanocrystallite–electrolyte interfaces due to spin–orbit coupling.

## Introduction

Porous nanophase materials with electrochemically induced tunability of properties [1] have become a topic of growing research interest in the past few years. Studies on the tunability of mechanical (e.g., [2–4]), electrical (e.g., [4–9]) and magnetic (e.g., [9–14]) properties have been presented for various materials. Besides field-induced tuning due to the accumulation of excess charges at the electrode–electrolyte interface of nanophase materials, the physical material properties may also be changed by electrochemical surface reactions, as for instance documented for nanoporous Au [6]. Based on tunability studies of the electrical resistance of porous nanocrystalline Pt [5] or nanoporous Pt [7] and of the magnetic moment of porous nanocrystalline Pd [10], the present work aims at a direct comparison of electrical resistance and magnetic moment with respect to in situ electrochemical oxidation by using porous nanocrystalline Pt as a model system. These different electronic properties represent an ideal combination to provide a deeper understanding of the underlying charge-related processes since both properties are expected to respond differently on charging and chemical modification. The studies make use of a specifically designed electrochemical cell that allows in situ magnetic studies in a SQUID magnetometer under electrochemical control [12].

## Experimental

Porous nanophase platinum samples were produced from commercially available platinum powder (Platinum Black, 20–40 m^2^/g, Chempur GmbH). For resistance measurements, 104 mg of Platinum Black were compacted into a PTFE groove with embedded electrical contacts similar to our previous work [5], improved by adding a fifth wire providing an independent contact for electrochemical charging (further referred to as sample Pt_ER_). For magnetic measurements, 17.8 mg of the powder were compacted to a cylindrical pellet, which was carefully wrapped by a gold wire, applying an electrical contact (further referred to as sample Pt_SQUID_).

All measurements were carried out at ambient temperature in a 1 molar aqueous solution of KOH. Resistance measurements were performed in a standard electrochemical cell with a PGZ-100 potentiostat (Radiometer Analytical). The Pt_ER_ sample served as the working electrode and was charged via the Pt wire contacted to the center of the sample. Porous carbon fabric and a commercial Ag/AgCl (sat. KCl) electrode (Radiometer Analytical) were used as counter and reference electrode, respectively. The electrical resistance was measured in a four-point geometry with a Keithley 2400 multimeter using the outer contact pair for current supply and the inner contact pair for voltage measurement. SQUID magnetometry was performed in a MPMS-XL-7 device (Quantum Design) at a constant magnetic field of 5 kOe upon in situ electrochemical charging with an Autolab PGSTAT128N potentiostat (Metrohm). The magnetic measurements were performed in a miniaturized electrochemical cell by using porous carbon fabric and a gold wire as counter and quasi-reference electrode, respectively, similar to our setup presented recently [12]. In the present improved setup a long borosilicate glass NMR tube (Wilmad-LabGlass, length 17.78 cm, diameter 4.96 mm) was used as the electrolyte container. By this means, the part filled with electrolyte extended well beyond the SQUID detection coils, and the counter electrode was located outside the coils, so that neither the electrolyte nor the counter electrode caused changes of the SQUID signal while scanning the tube through the detection coils. In the following, all potential values are given relative to Ag/AgCl, i.e., the data taken with the Au quasi-reference electrode are converted to corresponding values relative to Ag/AgCl. As reference values for the resistance and the magnetic moment, the initial values *R*_0_ and *m*_0_ of the respective measurement were used.

## Results

**Figure 1:**
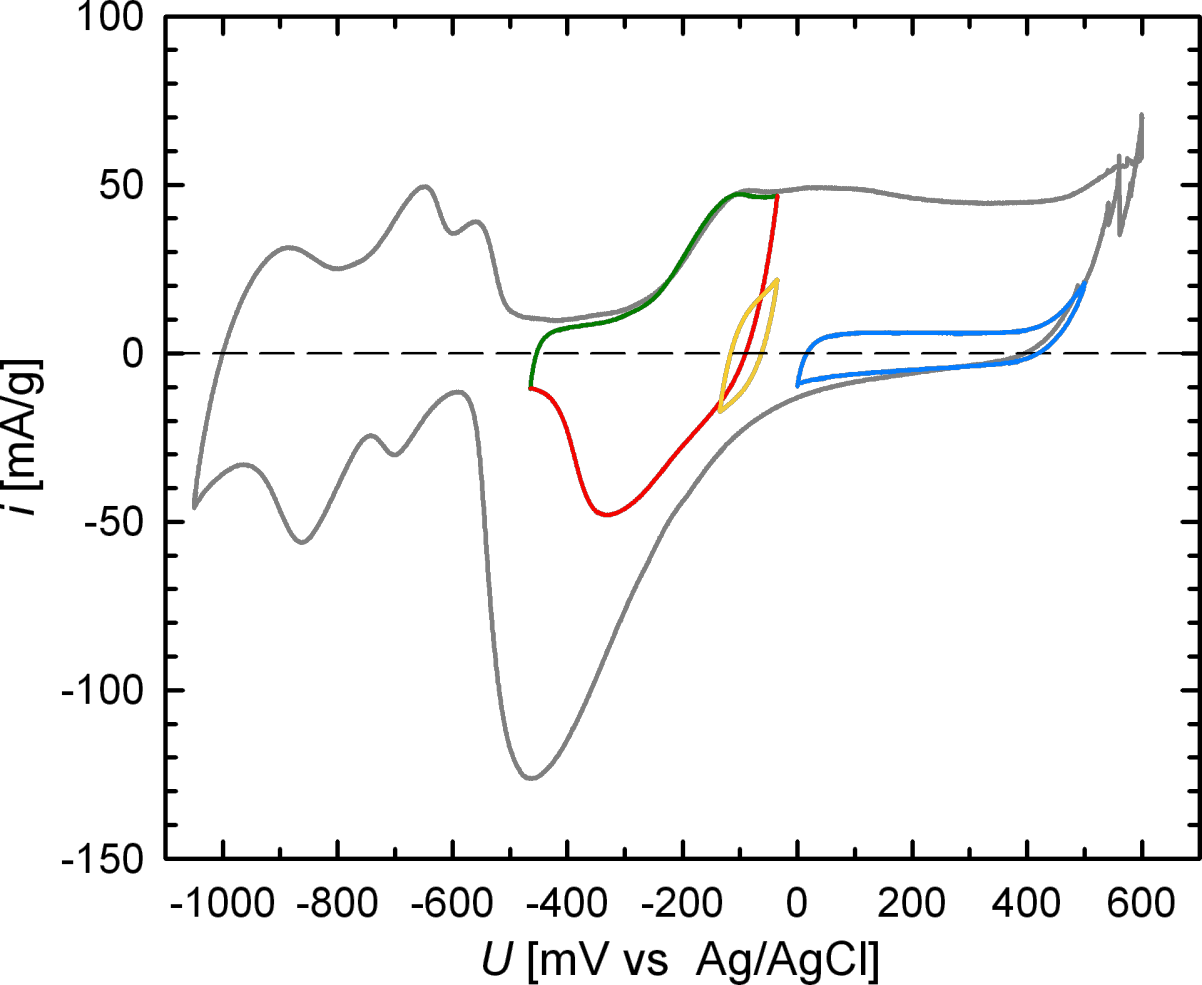
Steady-state cyclic voltammograms (CVs) of porous nanocrystalline Pt (sample Pt_ER_) measured at a scan rate of *v* = 0.5 mV/s in different potential ranges from −1050 mV to +600 mV (gray), −465 mV to −35 mV (green, red), −135 mV to −35 mV (yellow), 0 mV to +500 mV (blue) in 1 M KOH.

Charging was performed by the electrochemical methods of cyclic voltammetry, with a constant scan rate of 0.5 mV/s, or chronoamperometry (CA). Prior to the measurements the electrode was activated by repeatedly cycling between the oxygen- and hydrogen-evolution regimes. A typical cyclic voltammogram (CV) taken for sample Pt_ER_ is shown by the gray curve in Figure 1. As the potential is varied with triangular characteristics, the CV curve is run through clockwise. The behavior well-known from the literature (e.g., [15–16]) can be discerned, i.e., upon anodic scanning, the peak system due to hydrogen desorption (−950 mV to −550 mV), the double-layer region characterized by low charging currents (−500 mV to −300 mV), as well as the shoulder and plateau of oxygen adsorption/sample oxidation (−200 mV to 600 mV) followed finally by O_2_ evolution. In the opposite direction, oxygen remains on the surface until the desorption peak is reached (extremum at −460 mV), followed by the region of hydrogen adsorption (−650 mV to −1050 mV), and finally by the onset of H_2_ evolution.

The colored CV curves in Figure 1 characterize the various potential regions, which will be discussed in the following with respect to charge-induced variations of the electrical resistance and the magnetic moment. Each CV is located within the oxygen-governed regime and shown in steady state, which means that subsequent cycles perfectly superpose each other, and thus, identical electrochemical processes take place in all cycles. The CV between −465 mV and −35 mV characterizes the formation (anodic direction, green line) and subsequent removal (cathodic direction, red line) of a thin oxygen layer to an extent of less than one oxygen atom per Pt surface atom [16]. This layer is modified without being entirely removed in the narrow CV-regime between −135 mV and −35 mV (yellow line). The CV in the higher potential regime (0 mV to +500 mV, blue line) corresponds to the state of further strong oxygen adsorption.

**Figure 2:**
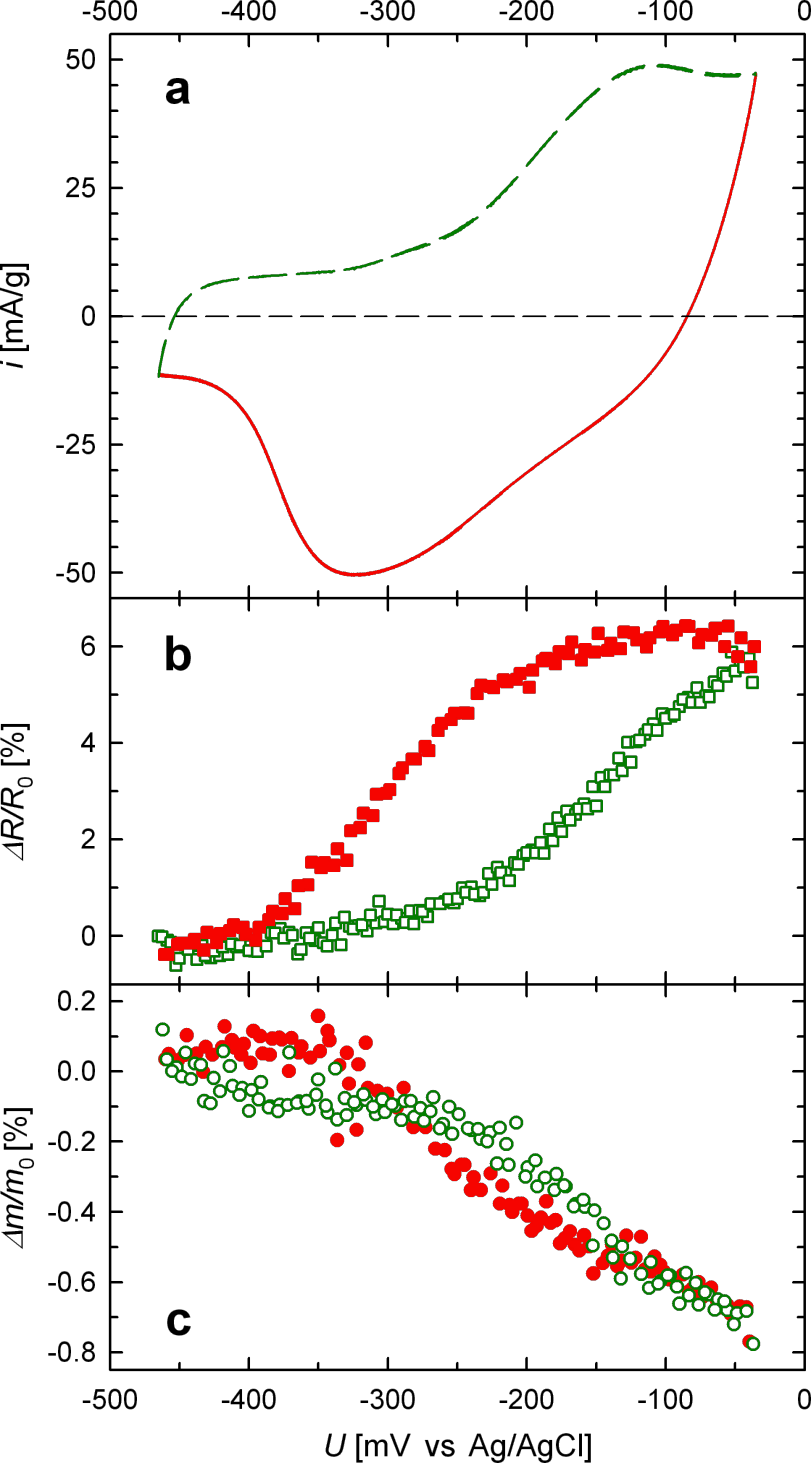
Relative variation of resistance (Δ*R*/*R*_0_, b) and magnetic moment (Δ*m*/*m*_0_, c) of porous nanocrystalline Pt upon electrochemical CV-cycling in 1 M KOH between −465 mV and −35 mV. (a) CV measured for sample Pt_ER_. Measurements of *m* were performed at 5 kOe. Anodic scan indicated by green dashed line and green open symbols. Cathodic scan indicated by red solid line and red full symbols. To demonstrate the good reproducibility and reversibility two cycles are shown in each plot.

The CV between −465 mV and −35 mV (green and red) is shown in more detail in Figure 2a along with the corresponding variation of the electrical resistance (b) and of the magnetic moment of the platinum samples (c). The green- and red-colored data in (b) and (c) again indicate the anodic and cathodic scan direction, respectively. In each plot two subsequent voltammetric cycles are plotted, which can hardly be discerned, illustrating the good reversibility of the measurements in the steady-state CV. The electrical resistance *R* reversibly increases upon positive charging, exhibiting a total variation Δ*R*/*R*_0_ of about 6% (Figure 2b). The variation of *R* with the potential *U* exhibits a pronounced hysteresis, which indicates a high sensitivity of *R* with respect to superficial oxygen adsorption. This sensitivity is also clearly reflected by the steep increase or decrease of *R* right in the CV regime where oxygen adsorption or desorption takes place, respectively. Taking a closer look at the high potential edge, a further increase in *R* can be observed when the scan direction is reversed (start of red-colored data) as the charging current *i* is still positive. When *i* finally changes its sign at about −75 mV, the resistance also starts to decrease. Thus the *R*-variation is proportional to the transferred charge as presented in more detail below (Figure 4). The magnetic moment *m*, on the other hand, decreases (increases) with increasing (decreasing) potential showing a total variation Δ*m*/*m*_0_ of approximately 1% (Figure 2c). The variation of the magnetic moment also changes strongly with the onset of oxygen adsorption, yet in contrast to *R*, the slope of the Δ*m*/*m*_0_-potential characteristic remains at a high value in the entire oxygen adsorbed regime, reversing its sign immediately at the high potential edge. This indicates a correlation to the capacitive charging currents (i.e., the electric field), which also reverse sign directly with the potential scan direction, explaining the lack of hysteresis for Δ*m*/*m*_0_.

**Figure 3:**
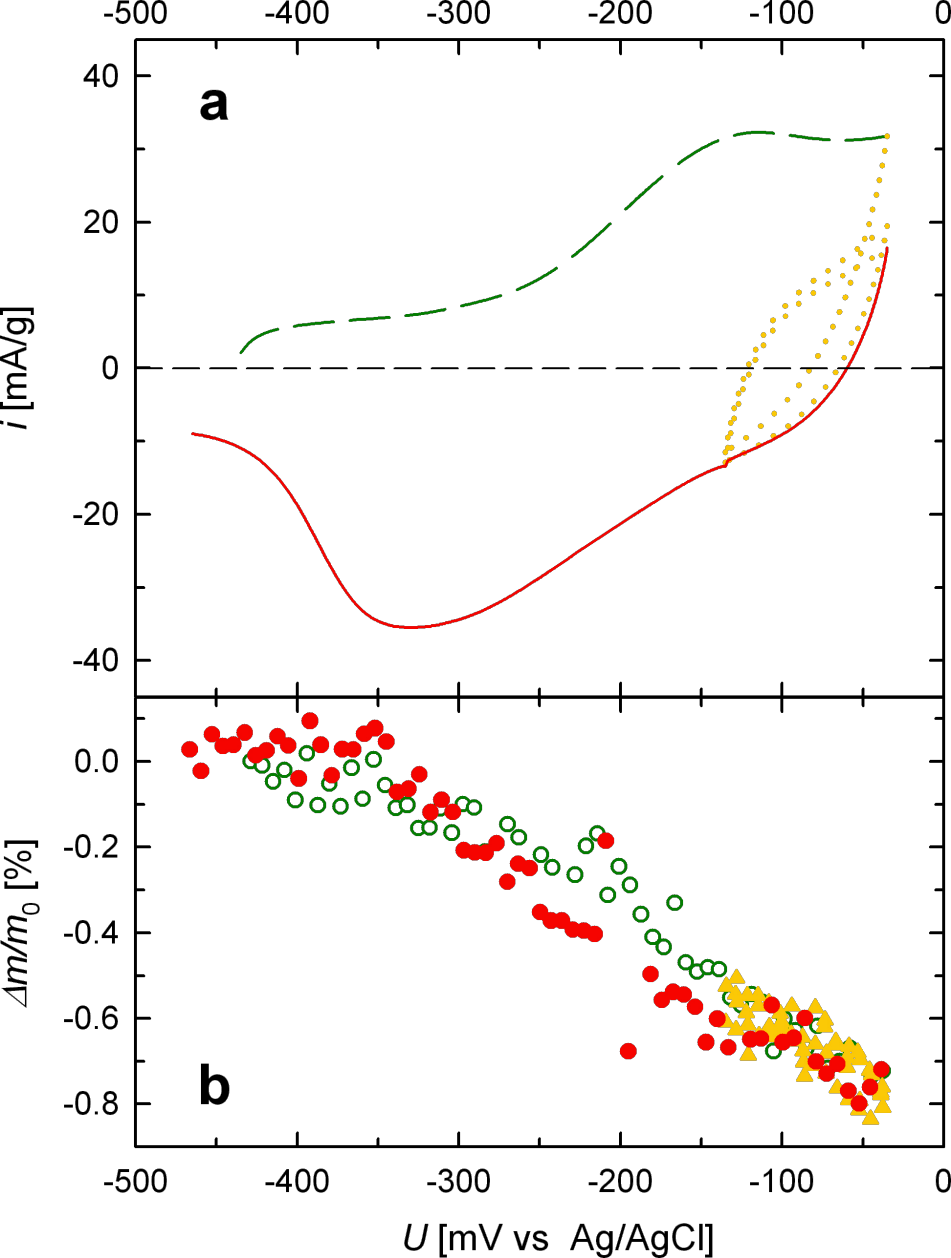
Relative variation of magnetic moment (Δ*m*/*m*_0_, b) of porous nanocrystalline Pt upon electrochemical oxidation (−435 mV to −35 mV, green: dashed line, open circles), electrochemical cycling between −135 mV and −35 mV (yellow: dotted line, full triangles), and electrochemical reduction (−35 mV to −465 mV, red: solid line, full circles). (a) CV measured in situ at the identical sample (Pt_SQUID_). Measurements performed in 1 M KOH at 5 kOe.

The variation of *m* with applied potential has been studied in more detail by cycling in the potential range between −135 mV and −35 mV (see Figure 3). The cycling was performed after oxidation (green-colored data) and followed by reduction (red-colored data), similar to the measurement in the potential range between −465 mV and −35 mV. The same variation Δ*m*/*m*_0_ with potential is observed for the range between −135 mV and −35 mV, irrespective of whether the CV cycling is limited to this regime (yellow-colored data in Figure 3) or whether the potential is scanned through the entire regime of electrochemical oxidation (green-colored data) and reduction (red-colored data). Since in the narrow CV regime the adsorbed oxygen remains on the sample, this leads again to the conclusion that the variation Δ*m*/*m*_0_ is more directly governed by the potential (i.e., the electric field) than by the superficial adsorption or desorption of oxygen. In contrast to these findings for *m*, no significant variation in *R* can be observed in this narrow potential range (data not shown).

**Figure 4:**
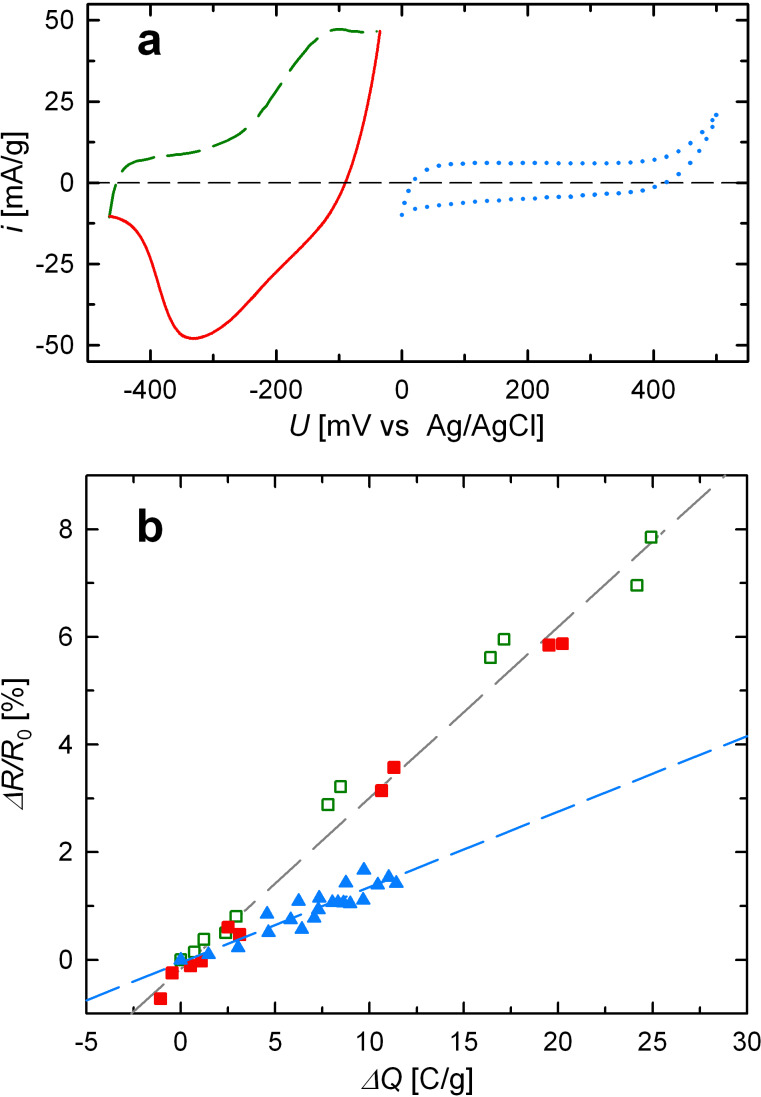
Relative variation of electrical resistance Δ*R*/*R*_0_ of sample Pt_ER_ upon adsorption (green: dashed line, open squares) and desorption (red: solid line, full squares), and upon charging in the regime of strong oxygen adsorption (blue: dotted line, full triangles) in 1 M KOH. (a) CVs measured in the respective potential ranges. (b) Variation of Δ*R*/*R*_0_ with charge Δ*Q* imposed upon chronoamperometry (CA) in the potential regimes shown in (a).

The dependence of Δ*R*/*R*_0_ on the applied charge was investigated using the electrochemical method of chronoamperometry (CA), where a correction of the leak current is possible, allowing for a higher accuracy of charge determination. Two different potential regimes were investigated, which are illustrated by cyclic voltammograms in Figure 4a. The regime between −465 mV and −35 mV, which is identical to that applied in the CV-mode measurement (Figure 2b), characterizes adsorption and desorption, whereas the regime between 0 mV and +500 mV corresponds to the steady state of strong oxygen adsorption (blue). In each potential region, CA measurements were performed in consecutive step intervals of 86 mV (green/red) or 100 mV (blue). After an equilibration time of 60 minutes at a given potential, the transferred charge Δ*Q* per sample mass, relative to the first measurement point in the respective regime, was determined from the monitored charging current and the electrical resistance was measured. The data Δ*R*/*R*_0_ in dependence of Δ*Q* obtained in this way are presented in Figure 4b. Again, two successive cycles are shown in each region, to illustrate the reversibility of Δ*R*/*R*_0_. The regime of adsorption/desorption (green, red) is characterized by a high charge coefficient [(Δ*R*/*R*_0_)/(Δ*Q*)]_Chem_ = 0.31 %·(C/g)^−1^. The measurements performed in this regime allow for a comparison of the CA- (see Figure 4b) and CV-mode (see Figure 2b), which yield self-consistent results, both indicating a strong sensitivity of *R* to the surface modifications. In the regime of strong oxygen adsorption (blue), besides a significantly smaller total variation of Δ*R*/*R*_0_, also the relative dependence on Δ*Q* is significantly weaker as reflected by a reduced value [(Δ*R*/*R*_0_)/(Δ*Q*)]_Ox_ of 0.14 %·(C/g)^−1^.

## Discussion

For the discussion of the present results, first of all, it has to be pointed out that the voltage-induced responses of the magnetic moment and electrical resistance, although nicely correlated, exhibit distinctly different behavior as outlined above. As discussed earlier neither the variation of the magnetic moment nor that of the resistance can simply be attributed to a variation Δ*n*/*n* of the charge carrier density upon electrochemical charging [7,10]. A simple Δ*n*/*n*-dependence for both effects can further be excluded, because for that, an equal magnitude of variation |Δ*R*/*R*_0_| = |Δ*m*/*m*_0_| would have to be expected. The obviously weaker sensitivity of the magnetic moment to the formation and removal of a thin oxygen layer, compared to the electrical resistance, can yet be understood by looking more closely at the different magnetic and electronic responses of the metal–electrolyte interface upon adsorption and desorption of oxygen species.

On the one hand, the increase in *R* with increasing potential can be understood as being dominated directly by surface effects, in good agreement with previous results [5,7]. Besides a reduced charge carrier density due to positive charging [17] and a shrinking conductor cross-section of Pt in favor of a superficial formation of platinum oxide with ongoing oxidation, the electrical resistance is strongly affected by charge-carrier scattering processes at the metal–electrolyte interface [5–7]. In the potential regime indicated by the green/red curves (Figure 2a and Figure 4a), oxygen adsorption takes place at a clean platinum surface. Since the ion transfer in this regime corresponds to less than one oxygen atom per platinum surface atom [16], each adsorbed ion may effectively act as an additional charge-carrier scattering center at the platinum surface. Therefore, the resistance increases strongly in this regime and, moreover, is directly related to the transferred charge, which reflects the amount of adsorbed oxygen.

Compared to the preceding regime of oxidation and reduction, the blue-colored regime (Figure 4a) denotes a steady-state condition well above the oxygen desorption peak where reversible charging is performed entirely within the oxygen adsorbed steady state without removing it. Since the corresponding currents in this steady-state CV are similar to that of the double layer regime of platinum (see Figure 1), the charge transfer in this regime can be considered as “double-layer-like” charging of a previously oxygen-covered surface. This results in weaker response of the resistance, because charging does not directly affect the underlying platinum surface. This explains the reduced charge coefficient in this regime (Figure 4b).

It is worth mentioning that, compared to the present case of cluster-assembled porous Pt, the charge-induced resistance variation of nanoporous Pt prepared by electrochemical dealloying in addition showed a sign inversion at high potentials [7]. This different behavior has to be assigned to the strongly different types of surface states originating from the preparation, which is a chemical reduction process for the commercial Pt powder, whereas dealloying takes place under strongly oxidizing conditions. In fact, as shown by Viswanath et al. [18], strongly differing surface states may respond rather differently with respect to charging. Regarding dealloyed nanoporous Pt, the sign-inversion of the charge-induced resistance variation was associated with the semiconducting behavior of the partially oxidized surface [7].

The electrochemical tunability of the magnetic moment is more than one half of an order of magnitude lower than that of the electrical resistance. The magnetic response is less sensitive to charging since the macroscopic magnetic moment of nanocrystalline Pt represents a volume average of the interior and surface region of the nanocrystallites, only the latter part being affected by charging. In contrast to that, the electrical resistance, which is governed by interfacial charge-carrier scattering, selectively probes the crystallite–electrolyte interfaces. The observed decrease of the magnetic moment of nanocrystalline Pt with electrochemically induced oxygen adsorption nicely fits with earlier studies of the influence of chemisorbed oxygen on the magnetic susceptibility of Pt [19]. The trend of decreasing magnetic moment is also supported by recent DFT calculations according to which the density of states at the Fermi level of Pt monotonically decreases with increasing oxygen coverage [20]. This also demonstrates that a simple picture of voltage-induced filling or depletion of rigid electronic bands fails, because within such a picture positive charging, i.e., extraction of electrons from the nearly filled d-band of Pt would give rise to an increase of the density of states rather than to a decrease.

Although oxygen adsorption causes a decrease of the magnetic moment, it may not fully account for the observed voltage-induced variation of *m*. In fact, in contrast to the voltage-induced variation of *R*, the Δ*m*/*m*_0_–*U* behavior shows no hysteresis (Figure 2c) but, on the other hand, *m* changes also in the narrow potential regime between −135 mV and −35 mV where oxygen is not removed from the surface (Figure 3). This indicates that the variation Δ*m*/*m*_0_ is governed by the electric field at the nanocrystallite–electrolyte interface. As well known for surfaces, the asymmetric potential drop at a surface causes strong spin–orbit coupling with mobile spins (Rashba effect) [21]. This is reflected by the formation of an effective magnetic field due to unbalanced orbital currents, which polarizes the electron spins. Since this Rashba spin–orbit coupling varies with an applied electric field *E* [21–22], this may give rise to an *E*-dependence of the magnetization, directly. In addition the Rashba effect is also associated with magnetic surface anisotropy [22]. The *E*-dependence of the latter may also cause variations of the magnetic moment in nanophase systems according to recent theoretical studies of Subkow and Fähnle [23]. Spin–orbit coupling in combination with potential-dependent surface stress or charge-induced relaxation of the outermost atomic layer may also influence the magnetization, as suggested by Ghosh [14]. More detailed in situ electrochemical SQUID studies, in particular measurements in dependence of the applied magnetic field, will be necessary in order to further elucidate the chemical or/and electric-field-induced character of the Δ*m*/*m*_0_–*U* behavior.

## Conclusion

In conclusion, the variations of the electrical resistance and magnetic moment upon electrooxidation show similar trends but differ in detail, since the former is due to charge-carrier scattering processes at the metal–electrolyte interfaces, whereas the latter seems to be governed by the electric field at the nanocrystallite–electrolyte interface. Consistent results for the resistance variation were found by chronoamperometry and cyclic voltammetry. The combination of SQUID magnetometry and in situ cyclic voltammetry opens up attractive potentials for studying nanophase materials under full electrochemical control.
